# Natural Products as a Source for New Anti-Inflammatory and Analgesic Compounds through the Inhibition of Purinergic P2X Receptors

**DOI:** 10.3390/ph6050650

**Published:** 2013-04-29

**Authors:** Rômulo José Soares-Bezerra, Andrea Surrage Calheiros, Natiele Carla da Silva Ferreira, Valber da Silva Frutuoso, Luiz Anastacio Alves

**Affiliations:** 1 Laboratory of Cellular Communication, Oswaldo Cruz Institute–FIOCRUZ, Rio de Janeiro 21040-360, Brazil; 2 Laboratory of Immunopharmacology, Oswaldo Cruz Institute–FIOCRUZ, Rio de Janeiro 21040-360, Brazil

**Keywords:** natural products, phytotherapy, purinergic receptors, inflammation and pain

## Abstract

Natural products have reemerged in traditional medicine as a potential source of new molecules or phytomedicines to help with health disorders. It has been established that members of the P2X subfamily, ATP-gated ion channels, are crucial to the inflammatory process and pain signalization. As such, several preclinical studies have demonstrated that P2X2R, P2X3R, P2X4R and P2X7R are promising pharmacological targets to control inflammatory and pain disorders. Several studies have indicated that natural products could be a good source of the new specific molecules needed for the treatment of diseases linked to inflammation and pain disorders through the regulation of these receptors. Herein, we discuss and give an overview of the applicability of natural products as a source to obtain P2X receptors (P2XR) selective antagonists for use in clinical treatment, which require further investigation.

## 1. Introduction

For thousands of years, natural products derived from plants, animals and microorganisms have been used as treatments for human diseases. Knowledge of the medical use of natural products has been transmitted from generation to generation over the years and has incorporated in several different cultures [1]. Their properties piqued scientific curiosity by the early nineteenth century with the discovery of morphine in 1805, after which various active molecules have been discovered for the treatment of many diseases directly or in the form of semisynthetic analogs [2]. Two notable examples are the antiplasmodial compounds quinine and artemisinin, which have been used clinically. Furthermore, they have also served as the basis of several semisynthetic antimalarial compounds [2]. Thereafter, several other molecules were discovered and used in the clinic, such as salicin, digitoxin and pilocarpine [2,3] to name a few. We can also cite antineoplastic agents, such as taxol and vincristine, since nearly 75% of them are derived from natural products [4]. Natural products have provided treatment for a range of disorders including inflammatory, parasitic, neurological, cardiovascular, metabolic, oncological and pain-related diseases [5]. In addition to providing a rich source of treatment possibilities, natural products also derive from a portion of the diverse of biological species in the world, of which there are estimated to be about 12.5 million according to the classic World Conservation Monitoring Center’s work [6]. Only between 10 and 15% of species have been exploited for therapeutic use. The key to capturing the potential of natural products will be in the ability to screen specific targets efficiently and to analyze many compounds simultaneously [5,7].

The WHO reports that treatments with herbal medicine or vegetable extracts are practiced by approximately 80% of the world´s population. Currently, phytotherapics represent an approximately $14 billion/year industry, about 5% of the current $280 billion/year market for medications. In this point, clear regional differences exist between developed and developing countries, where herbal products represent 25% and 80% of medications, respectively [8,9,10]. Among the 56% of currently prescribed synthetic drugs, 24% are derivatives from plant species, 9% are synthetic products modeled from natural products, 6% are extracted directly from the plant species and 5% of animal origin [11].

However, the scope of what natural products can offer human medicine is far from being realized. The estimated total of existing species is between 350,000 to 550,000, of which less than 20% having been investigated for medicinal potential [8]. Brazil, for example, has around 10% of the worldwide flora and has less than 1% of its plant species have been investigated from a chemical and/or pharmacological point of view [12].

Inflammation is a growing area of investigation and target for intervation in a multitude of disease conditions. One pharmacological target in the area is P2XR, which are important receptors in the modulation of inflammation and pain. Herein, we discuss the natural products which have shown antagonistic activities on P2XR and their relevance for future research in alternative medicines [13].

## 2. Natural Products and Their Potential as Modulators of the Inflammation and Pain Related P2X Receptor

**Figure 1:**
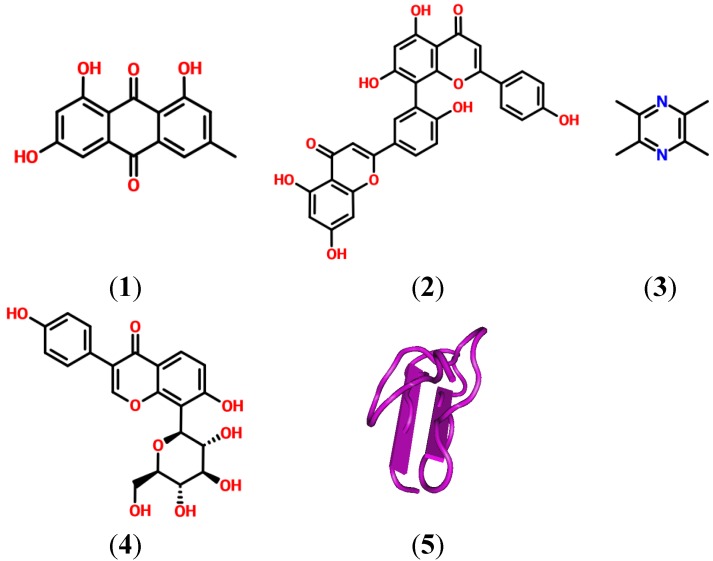
Molecules from natural products with analgesic activity through P2XR antagonism [18]: (**1**) Emodin; (**2**) Amentoflavone; (**3**) Ligunstrazine; (**4**) Puerarin and (**5**) Purotoxin-1 (PDB: 2KGU).

Natural products have recently been reported as showing antagonistic properties on the P2R receptors [14]. Several of those that were found to be related to analgesic or anti-inflammatory activity on P2XR are presented in Figure 1.

The importance of this finding is the role of some of the P2R receptors in inflammation and pain, more specifically the P2XR family, which is one of the two subclasses of the P2 receptors (P2R). The P2XR are ionotropic and have ATP as their main agonist. Among the seven subtypes of P2XR, the types that are most related to the development or control of pain states, are the P2X3R, the heteromeric P2X2/3R, P2X4R, and the P2X7R [15,16,17].

There are currently about 90 published papers that describe the role of P2XR in pain and/or inflammation. The role of P2XR is chiefly based upon its expression in the central and peripheral terminals and spinal cord [15]. Antagonists of this receptor show a significant analgesic effect, and an agonist (ATP) demonstrates a hyperalgesic action [16].

P2X3R and the P2X2/3R are expressed selectively in the sensory system, at the peripheral afferent fibers [19]. Damaged cells release stimuli molecules, such as glutamate or ATP (the principal agonist of these receptors), and initiate noxious signals, leading to neuronal excitability [16,17] and pain [19]. The homomeric P2X3R and its heteromeric P2X2/3R are also related to the inflammatory response, however it remains unclear whether the mechanism of the development of the inflammatory hyperalgesia is dependent on inflammatory cytokines [20] or the release of PGE2 and sympathomimetic amines causing a translocation of PK Cepsilon [21] or the release of bradykinin or whether it has no correlation with the release of cytokines, PGE2 or dopamine [22].

Several recent studies have found that natural products are able to inhibit the P2X3R mechanism. A recent work [23] using a rat model demonstrated an analgesic effect on formalin-induced pain through the antagonism of the P2X3. An herbal product used in Chinese medicine called ligustrazine (tetramethylpyrazine), an alkaloid derived from *Ligusticum wallichii*, caused an inhibition of the membrane depolarization induced by ATP in dorsal root ganglion neurons. Another study [24] confirmed this compound’s capability to inhibit ionic currents induced by ATP in dorsal root ganglion neurons through antagonistic activity on P2X3R. It was also found that its effect was not selective, since it also acted on PKC phosphorylation.

The analgesic effect of tetramethylpyrazine was also found on neuropathic pain through the blockage of the primary afferent transmission by P2X3R activation [25], and related to the compounds effect on pain transmission after a burn as also being through a P2X3R mechanism [26]. Another natural product known as puerarin was also found to have an inhibitory effect on burn pain hyperalgesia through inhibiting the upregulation of the P2X3R protein expression in the dorsal root ganglion neurons [27], with similar analgesic effect on neuropathic pain through the same mechanism elicited in the hyperalgesia context [28].

Similiarly, emodin, an anthraquinone obtained from rhubarb (*Rheum officinale* Baill) extract demonstrated analgesic activity not only on neuropathic pain through antagonism of the P2X3R expressed in the primary sensory neurons [29], but also had antagonistic activity on the rat P2X7R transfected in HEK-293 cells [30]. Purotoxin, a peptide isolated from the venom of the Asian spider *Geolycosa* sp., also showed a potent and selective antagonist effect on P2X3R, inhibiting the ionic current in rat neurons and demonstrating an analgesic effect on inflammatory pain [31,32].

While P2X4R has been linked to acute and chronic pain by several authors [33,34,35], its role in the development of both neuropathic and inflammatory pain was recently proposed. In the periphery, when the P2X4R present in macrophages is activated, these cells release prostaglandin E2 (PGE2), through a p38 MAPK (mitogen-activated protein kinase) pathway; this prostaglandin leads to a hipersensitivity of the peripheral nerves, causing inflammatory pain. In the CNS, where the microglia express P2X4R, when activated, leads to an increase in the intracellular calcium activating the MAPK pathway with the release of BDNF (Brain-derived neurotrophic factor) which then acts on the release of GABA (a hyperalgesic molecule) by gabaregic neurons. The inhibition of the production of BDNF through treatment with an antagonist to P2X4R (TNP-ATP) was observed *in vitro* [35]. Unfortunately, there is a lack of studies of new synthetic or natural compounds with activity on the inflammatory and pain disorders that could benefit through the blockage of this P2X4R receptor. Still Hernandez-Olmos *et al*. [36] demonstrated the activity of N-substituted phenoxazine and acridone derivatives on the calcium influx in 1321N1 astrocytoma lineage cells and cells transfected with the human P2X4R, showing the possible application of these compounds in the nociception context. Also, the participation of P2X4R in the inflammatory context is related to the co-expression with the P2X7R, and it mediates the inflammatory functions related to P2X7R through the release of cytokines (*i.e.*, IL-1beta) and cell death signaling through a calcium influx [37]. Its receptor also mediates the release of PGE2 via MAPK activation, which participates in the maintenance of a chronic inflammatory response [35].

Finally, P2X7R is expressed in the CNS, microglia, Schwann cells and astrocytes [16]. The correlation between P2X7R and pain is well documented, with approximately 94 published papers. The mechanism includes either the release of ATP or glutamate [16] through damage to the neuronal periphery cells, the P2X7R expressed in microglia, promotion of the potassium efflux and activation of the inflammasome complex the release of IL-1beta and superoxide anions, with the nitrite peroxide acting on pain maintenance through the neuronal periphery pathway [38].

Several inflammatory diseases are related to P2X7R activation, most notably rheumatoid arthritis, which has earned attention and scientific efforts in the application of new treatments through the inhibition of this receptor [38]. The functional role of the P2X7R in the inflammatory event is well documented [39] as it is an important mediator in the expression and release of some cytokines and inflammatory mediators, such as IL-1beta [40,41], IL-1α [42], IL-2 [43], IL-4, IL-6, IL-13, IL-18 [44,45], TNF-α [46], Nitric Oxide (NO) [47,48] and superoxide anions [49]. Its key role makes it an important anti-inflammatory target [50], not to mention a potential therapeutic pain target, and it has received attention from pharmaceutical companies that are evaluating clinical trials based on it. Recently the antagonistic effect of bisflavonoids from the methanolic extract fractions of *Rheedia logifolia* on the P2X7R has been found to promote analgesic action on inflammatory pain [51].

## 3. Could an Antagonist to P2XR be Useful in the Treatment of Inflammatory and Pain Disorders?

The search for an effective treatment for inflammatory and pain disorders has been the main aim of many scientific groups and pharmaceutical companies worldwide. As these diseases afflict a great portion of the worldwide population, purinergic receptors have come to the attention of the scientific community for their participation in the inflammatory and pain pathways, classifying them as potential therapeutic targets for these diseases.

Among all P2R receptors, the P2X3R, P2X2/3R, P2X4R and P2X7R are the most implicated in the development and maintenance of the inflammatory and pain pathways. They have become the target for evaluation in *in vitro* tests, drug trials, clinical trials and synthesis of antagonist compounds based on molecular docking. Currently, while there are many trials being evaluated using synthetic compounds to resolve or attenuate the signs and symptoms of inflammatory and pain conditions, there are none yet of natural products, and these represent a new and unexplored research approach.

There have been several new synthetic compounds developed for both the P2X3R and P2X2/3R and P2X7 receptors. For the former, the most notable synthetic compounds are AF-353, developed by Roche Pharmaceuticals [52], and A-317491, developed by Abbott Laboratories. For the latter, candidates for a potential antagonist for P2X7R with selective action and possible clinical applicability [53] have arisen, such as the disubstituted tetrazoles, the cyanoguanidines A-438079, A-740003 and A804598 with action on hP2X7R (human)/mP2X7R (mouse) and rP2X7R (rat) [54].

A recently published study that demonstrated the efficacy and tolerability of the compound CE-244,535 (a selective P2X7R antagonist *in vitro* and *in vivo*) in humans with rheumatoid arthritis, showed no significant increase in efficacy in comparison to the current treatment [55].

Natural products have begun to open up new possibilities for inhibition and intervention in inflammatory and pain pathways. There is a clear need to investigate further the preliminary findings that have recently emerged and investigate more profoundly the possibilities and potential of natural products for the inhibition of these receptors and their applicability to the treatment of diseases related to these receptors.

## 4. Conclusions

While the participation of P2XR in the development of inflammatory and pain disorders is already known, they continue to gain much attention for their importance as a therapeutic target. With emerging evidence of natural products showing potential therapeutic properties in the regulation or influence of these receptors it has brought to surface a new player in this search. Beyond any possible pharmaceutical treatments they have the potential to offer insight into the mechanisms that are at work within these conditions as well as to possibly identify novel approaches based upon the natural properties of the substances. Inasmuch as the research about natural products and P2XR is still in the beginning, it has begun to make promising results demanding more attention and efforts on the development and generation of a possible byproduct that can be useful in the future as a phytotherapy treatment of these diseases.
